# Towards an understanding of the impact of micro- and macro-manifestations of religiosity on climate change risk perception: a cross-national study

**DOI:** 10.3389/fpsyg.2026.1740305

**Published:** 2026-03-04

**Authors:** Richard Saunders, Marco Pomati, Nick Pidgeon

**Affiliations:** School of Social Sciences and School of Psychology, Cardiff University, Cardiff, United Kingdom

**Keywords:** christianity, climate change, cross-national, multilevel, psychology, religion, risk perception, sociology

## Abstract

**Introduction:**

This study examines how religion shapes climate change risk perception at individual and national levels across 28 countries, addressing gaps in cross-national research on religiosity and environmental attitudes.

**Methods:**

Using data from the ISSP Environment IV module (2023), we apply multilevel models to assess the effects of religious affiliation, religious practice, and national religious context, including majority religion, proportion of Christians, and national income.

**Results:**

Regular attendance at religious services is generally associated with lower climate change risk perception, while religious affiliation shows limited and country-specific effects. At the national level, predominantly Christian, especially Protestant, countries exhibit lower risk perception than Eastern-religious (Buddhism and Hinduism) majority countries. Cross-level interactions reveal an asymmetric role for religion based on national wealth, with religious affiliation influencing risk perception more significantly in lower-income countries.

**Discussion:**

The findings indicate that religiosity primarily influences risk perception through practice, while national religious contexts reflect enduring historical and institutional legacies. Evidence also indicates the effects of religiosity are, in part, contingent upon country-level material wealth.

## 1 Introduction

### 1.1 Purpose

Climate change represents an unprecedented challenge to human societies, with anthropogenic origins supported by multidisciplinary scientific consensus and accelerating global impacts emphasising the importance of a robust response. Climate change risk perception has also increased, though individual responses continue to vary substantially (Fagan and Huang, 2019; Capstick et al., 2015). Socio-psychological approaches have focused on the role of cognitive, affective, and socio-cultural factors (Van Der Linden, 2015), with sociological literature indicating that risk perception varies across sex, age, education, as well as attitudinal, ideological, and value orientations (Lee et al., 2015).

A variety of factors that shape climate change risk perception have been examined in the literature. These include sociodemographic characteristics, such as sex/gender (McCright and Dunlap, 2011; Bord and O’Connor, 1997), age (Whitmarsh, 2011; Poortinga et al., 2019), education level (Knight, 2016; de Bruine Bruin and Dugan, 2022), and income (Hornsey and Pearson, 2024). Other influential variables include political party affiliation (Kvaløy et al., 2012; Hornsey et al., 2018), perceived efficacy (Brody et al., 2008; Kellstedt et al., 2008; Capstick and Pidgeon, 2014), and value orientations (Corner et al., 2014)—though the explanatory power of these factors is highly variable across countries. Cognitive and emotional components are considered fundamental (Van Der Linden, 2015). Furthermore, experiences of extreme weather events have also been found to impact climate change risk perception (Spence et al., 2012; Dunlap and York, 2016; Lujala et al., 2015; Kvaløy et al., 2012; Shao and Goidel, 2016; Bergquist et al., 2019; cf. Brody et al., 2008; Frondel et al., 2017; Hoffman et al., 2022). Many of these elements are integrated into the climate change risk perception model (CCRPM), which explains approximately 40–70% of the variance in climate change risk perception, depending upon the country analysed (Van Der Linden, 2015; Castellini et al., 2024) and represents an important tool for policy development and dissemination.

Existing models of climate change risk perception often operate within implicitly secular frameworks or assume a secularising trajectory in society. As a result, they tend to overlook the ways that religious worldviews, identities, and institutions shape environmental concern. Yet, religious belief can be an important aspect of social identity and moral reasoning beyond political orientation, in poorer and richer countries, serving important social functions. This research also proposes a dual effect of religion, stemming from its functionalist role, as a stable epistemological framework that can offer a clear perspective of material and spiritual phenomena. However, its social and ideological rigidity can also place it in opposition to elements of alternative epistemological frameworks, such as scientific inquiry. Religiosity corresponds to the strength and certainty of beliefs and experiences, engagement with religious practice, and the level of community involvement, all emphasised as contributing factors (Holdcroft, 2006).

This study aims to understand the influence of religion on climate change risk perception. Our primary aim is to explore the effects of individuals’ beliefs and practices, as well as their impact from institutional and population-level perspectives. We first review the empirical literature on this topic and then propose a more cohesive theory of the effect of religion. We then test this theory on International Social Survey Programme data from 28 countries with varying religious characteristics. We carry out cross-country analysis to understand the impact of individual-level religiosity on risk perception and how this can vary substantially across countries, depending upon other national characteristics such as Gross Domestic Product (GDP) per capita and the majority religion.

### 1.2 Climate change risk perception

Climate change is a distinctive issue from several perspectives, with geographically uneven effects, meaning that an individual’s personal experience is, in many ways, reliant upon the environment in which they live. Furthermore, it partly relies on a robust conception of the future and awareness of historical trends and natural processes to fully grasp the issue, representing a challenge from a temporal perspective (Spence et al., 2012), and establishing it as a technical issue for which a substantial scientific knowledge base is necessary, particularly given that direct personal experience of such change is typically lacking. Nonetheless, risk perception can emerge from knowledge and experience of any one of these elements to a degree, and as such represents a complex analytical phenomenon in itself.

Farrokhi et al. (2020) highlighted three core dimensions of climate change risk perception: a mental health dimension with subcategories of emotion, mood, and personal experiences of risks; a cognitive dimension including information and understanding of the threat; and the interaction of imposed components, which pertains to cultural, social, economic, and political factors, including religion. However, as we argue below, the latter has received considerably less attention, partly due to a lack of a clear theory on how different types and levels of religiosity affect climate change concern.

### 1.3 Religious affiliation and attendance of religious services

Various elements of religiosity have been explored in cross-national research in relation to climate concern, such as the professed ‘importance of God’ and ‘frequency of prayer’ (Mostafa, 2016) and ‘religiousness’ (which asks respondents whether they consider themselves a religious person) (Felix et al., 2018). Zemo and Nigus (2021) utilised the following four measures of religion: attendance of religious services, importance of God, membership of religious organisations, and religiosity (the same measure as Felix et al. (2018) from the World Values Survey). These studies indicate that these are positively associated with climate concern and the notion that environmental protection should be prioritised over economic growth. These studies do not place much emphasis on the ideological distinctions between world religions, however, which likely shape individual, social, and cultural perspectives on environmental issues to a degree.

Research has also explored religiosity in single-country studies. Arbuckle (2017) explored the interaction of religion and politics in the USA, finding that climate concern is suppressed amongst democrats affiliated with Evangelical, Black Protestant, and Catholic religions, while Jewish democrats indicate higher concern than non-religious democrats. This emphasises the role of religious ideology as a moderating factor in the USA, where political affiliation is a central contributor to climate change perceptions, including risk perception (McCright and Dunlap, 2011). Pepper and Leonard (2016) found that religion directly affects Australians’ climate change beliefs, with scepticism higher amongst Pentecostal and small Protestant denominations than the general population. Pepper and Leonard (2016) emphasised the prevalence of dominion ideology, which refers to humanities’ dominion over nature, amongst these religious denominations as well as those noted from the USA (Tyson, 2021), echoing the emphasis placed upon anthropocentrism (a worldview that places human needs, interests, and authority at the centre, often prioritising sthem over ecological systems or non-human life) in Christianity (White, 1967). Nonetheless, it remains unclear whether the broad nature of the Christian tradition, which also places emphasis on humanity’s role as stewards of the earth (Shin and Preston, 2021; Michaels et al., 2021), leads to individual and denominational beliefs and related climate change concerns that vary across countries.

### 1.4 Religious majorities

Religious majorities represent another underexplored dimension of religiosity in cross-national research. The predominant religion in a country can offer distinct understandings of how religion impacts climate perceptions, over and beyond individual religious affiliation. The historical, cultural, and institutional influence exerted by major religions in different countries may be important for understanding contemporary climate discourse and perceptions amongst respective populations. This proposed influence on environmental and climate perceptions mirrors the historical development of capitalism, as outlined by Weber (1992). Weber argued that Protestant ideology played a foundational role in capitalism’s emergence. Over time, however, these religious values became institutionalised, eventually secularised, and embedded in scientific and economic structures—leaving their ideological origins largely forgotten (Black, 2015), yet still reflected in national differences (Desbordes and Munier, 2021; Trittler, 2017).

Recently, Tyson (2021) developed Progressive Dominion Theology (PDT) as a primarily cultural driver of climate change, arguing that anthropocentrism, an ideological perspective particularly present in Western, Christian countries, has resulted in changes to the global ecosystem at an unprecedented rate. This resonates with Skirbekk et al. (2020a) who argued that country-level religious differences may influence variations in climate behaviours in countries as they grow richer, such as energy use per capita (Skirbekk et al., 2020b).

Contemporary prevalence of religious affiliation may also lead to variation in climate change risk perception, which is distinct from historical institutional influence. Evidence indicates that more religious populations adopt less stringent climate policies (Shin and Preston, 2021; Sharma et al., 2021) and are negatively correlated with energy use (Skirbekk et al., 2020a). Chuvieco et al. (2016) showed that differences between countries based on the largest religious demographics (including non-religious) can impact environmental performance, with non-religious countries indicating the highest Environmental Performance Indicator (EPI) scores. Chuvieco et al. (2016) also rejected White’s (1967) theory that more anthropocentric religions will lead to worse environmental outcomes, due to evidence that Christian countries indicate higher EPI scores than other religions. However, this should be regarded with caution due to the lack of explanatory power for the majority religion when GDP and the Human Development Index (HDI) are included as measures. Skirbekk et al. (2020b) emphasised that variation in levels of religiosity and in the major religion itself across countries may incur distinct climate behaviours while they develop too.

### 1.5 Fundamentalism

Results regarding religious fundamentalism’s effects on environmental behaviours remain mixed (Chung et al., 2019; McCright, 2016), with little evidence indicating any effect on climate attitudes, including risk perception. These mixed findings may stem from the diverse manifestations of ‘fundamentalism’. While literalist beliefs often correlate with authoritarianism (Hunsberger, 1995), specific values, such as biospheric altruism, can still support pro-environmental attitudes (Chung et al., 2019). In the USA, fundamentalism has been shown to have deleterious effects on pro-environmental attitudes (Preston and Shin, 2022).

### 1.6 Wealth and religion

The influence of religiosity on climate change risk perception likely varies across countries, with national wealth emerging as a key moderating factor. Evidence suggests that more religious populations are generally found in less affluent countries, where religiosity is negatively correlated with GDP per capita, emissions, and energy use (Skirbekk et al., 2020a). This suggests that religious beliefs may shape climate attitudes differently depending on a country’s economic context.

One explanation for the limited exploration of religiosity may be the secularisation hypothesis, which posits that higher national wealth correlates with lower levels of religiosity (McCleary and Barro, 2006; Herzer and Strulik, 2013). This suggests that the observed relationship between wealth and climate change attitudes may already account for much of religion’s influence. However, there are several reasons to question this assumption. Firstly, literature has found contrasting results for climate change risk perception and national wealth. In Europe, higher GDP per capita is associated with higher awareness of climate change risks (Ergun et al., 2024). However, Echavarren et al. (2019) found mixed results across models. Kvaløy et al. (2012) found insignificant results. Similarly, results for the effect of country and household wealth on climate change concern are mixed, with both statistically significant negative relationships (Fielding et al., 2020) and positive relationships (Mayerl and Best, 2018). Hornsey and Pearson (2024) utilised HDI as a metric of national affluence, finding no significant relationship. Secondly, countries such as Israel, the USA, Singapore, Japan, and Italy maintain high levels of religiosity despite their relatively high national and household wealth, challenging the universality of the secularisation thesis.

## 2 Towards a more cohesive theory of religion

As shown above, the literature has explored the empirical relationship between environmental/climate perceptions and religiosity to some extent, yet attempts to develop theory have been primarily restricted to Christianity (Curry, 2008). The section develops, based on the existing literature, an expected ranking of climate change concern according to religious affiliation for comparative analysis of religions across the world. Although religiosity encompasses many dimensions, we focus here on the expected influence of religious tenets, summarised through the concepts of anthropocentrism, determinism, and eschatology, and religious behaviours such as service attendance and fundamentalism, in shaping climate change concern. Finally, we turn to the expected relationship between country wealth and religion.

### 2.1 Anthropocentrism vs. ecocentrism

The influence of anthropocentrism on climate change risk perception remains underexplored. Existing research suggests that biospheric values—often aligned with biocentric worldviews—are strongly associated with heightened climate change risk perception, more so than contextual factors such as response-knowledge or social norms (Van Der Linden, 2015; Pidgeon et al., 2003). Conversely, egoistic values, which emphasise self-interest and align closely with anthropocentric thinking, tend to correlate with lower risk perception, although this relationship requires further empirical clarification.

Anthropocentrism appears to be multi-dimensional in its effect. One dimension is egoism, which excludes both humanitarian and biospheric concerns, likely leading to reduced perception of climate risk. Another important dimension is short-termism—a prioritisation of immediate, tangible concerns (e.g., inflation, energy costs and security, stock market growth) over long-term environmental threats. This short-term orientation, observable across both egoistic and altruistic value frameworks, contrasts with future-oriented thinking, which has been linked to higher concern about climate change (Zhu et al., 2020). Motivated reasoning may reinforce short-termism, particularly among individuals driven by partisan or ideological identities (Bayes and Druckman, 2021), with religion potentially functioning in similar ways (Ezawa and Fagan, 2015).

Cognitive biases shaped by religious belief may also influence climate risk perception. For example, Dutch Calvinists exhibit attentional biases that favour local over global stimuli, potentially contributing to a focus on short-term or localised concerns (Colzato et al., 2008). While this cannot be generalised across global religious contexts, it suggests a mechanism through which religiosity may intersect with anthropocentric and short-termist orientations.

Climate change denial and scepticism represent further distinct pathways. Some anthropocentric individuals perceive human influence on the climate as inherently limited—a belief sometimes rooted in religious ideology—which may reduce belief in anthropogenic climate change (Carr, 2010; Schuman et al., 2018). Additionally, motivated reasoning driven by value conflicts can lead to denial as a means of resolving cognitive dissonance, particularly when climate action is seen as incompatible with other pressing social or political concerns (Bago et al., 2023).

**Figure 1:**
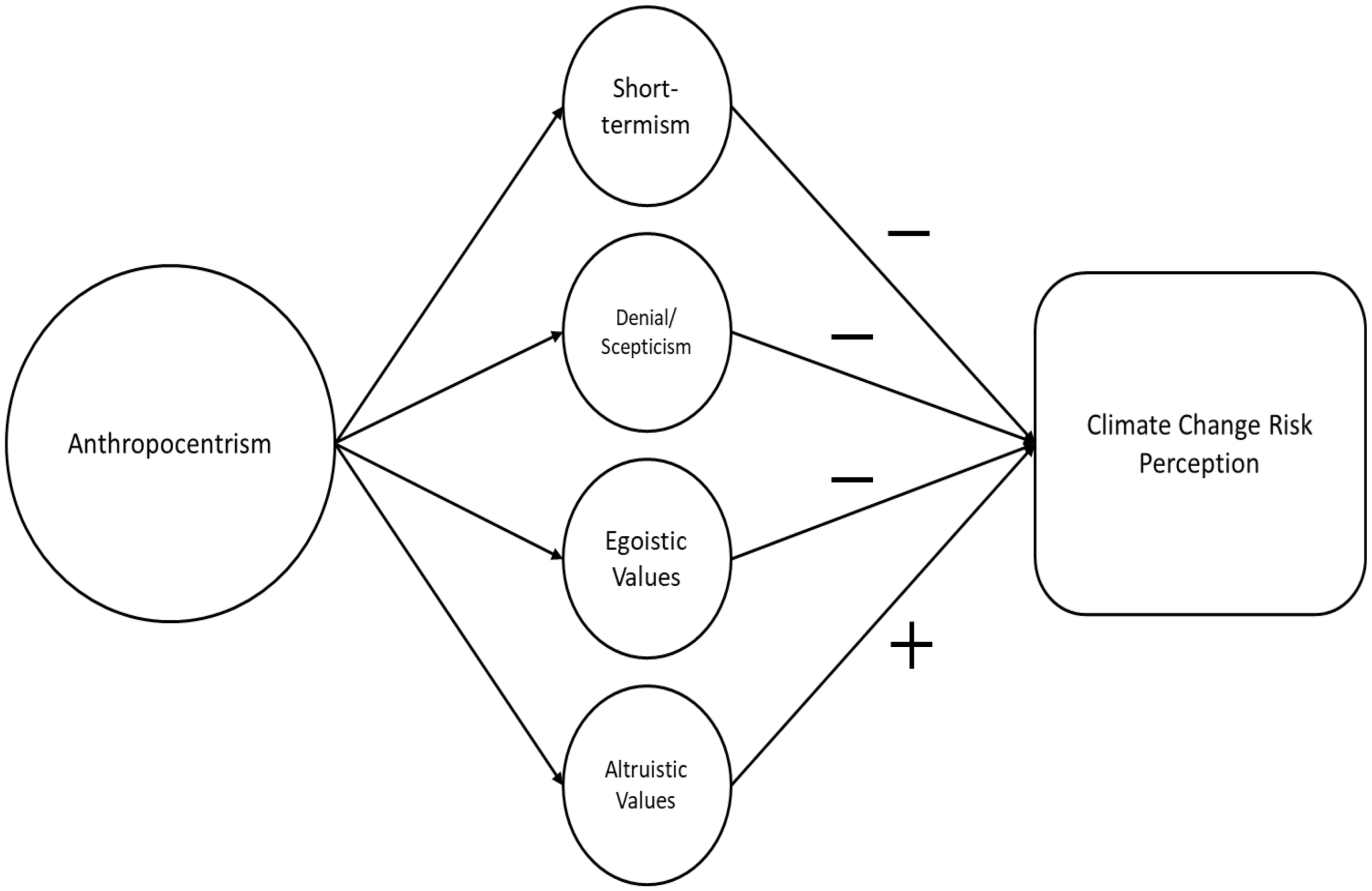
Components of anthropocentrism expected to affect individual’s climate change risk perception. Short-termism, denial/scepticism, and egoistic values indicate lower risk perception, while altruistic values indicate higher risk perception.

The mechanisms through which anthropocentrism influences climate change risk perception proposed here are as follows: (1) short-termism, (2) denial/scepticism, (3) egoistic values, and (4) altruistic values, with the latter expected to increase risk perception in our proposed model (Figure 1).

### 2.2 Free will and determinism

Religious perspectives on human agency—whether individuals have control over their own actions or global phenomena in general—may influence how said individuals perceive climate change risks. The extent to which religions prescribe agency or assign responsibility for environmental outcomes varies, with implications for climate-related attitudes (Curry, 2008). Deterministic views, where events are believed to be preordained by divine will, fate, or from the mechanics outside the control of the agent, i.e., electrochemical processes in the brain (Sapolsky, 2023), are contrasted with free will, where individuals are seen as capable of influencing outcomes as free agents. While philosophical debates continue to explore bridges between these perspectives, e.g., compatibilism (Pereboom, 2014), most scholars lean toward either a deterministic or free-will-based understanding (Wisniewski et al., 2022).

Deterministic religious beliefs can lower perceived climate risk. Farrokhi et al. (2020) reported that some individuals view climate events as divine will, reducing personal worry. Similarly, in low-lying Pacific Island communities, there has been some relocation to less vulnerable land in Australia: those who stay often maintain that God will protect them (Mortreux and Barnett, 2009; Swim et al., 2010). Kane and Perry (2024) found that belief in an interventionist God correlates with lower climate concern and lower perceived urgency for policy action in the USA. This is true for Muslims too, amongst whom belief in fate and predestination is widespread (Yilmaz et al., 2018; Pipes, 2015). Sunni Muslims in Turkey, for instance, show higher fatalistic determinism, though Islamic scripture also emphasises human accountability. Similar intra-faith variation exists in Christianity, with Orthodox Christians tending more toward fatalism than Protestants (Pipes, 2015), who emphasise individual responsibility (Weber, 1992).

Non-Abrahamic traditions also reflect complex views. In both Hinduism and Buddhism, karma implies moral causality but also raises questions about personal control, blending free will with determinism (Silvestre, 2017). Buddhism and Daoism often reject the notion of autonomous selfhood, suggesting a form of soft determinism (Repetti, 2014; Gier and Kjellberg, 2004; Ni, 1993). Daoism is antithetical to the Western or libertarian notion of free will; however, Daoist philosophy nonetheless promotes environmental responsibility by opposing human disruption of natural order (Xia and Schönfeld, 2011). This responsibility extends both towards and beyond individual humans, making this conception of agency particularly layered and complex.

Religion also shapes perceptions of self-efficacy regarding climate action. Morrison et al. (2015) found that religiosity affects beliefs in personal effectiveness, even after controlling for socioeconomic status. Some Christian literalists exhibit strong faith in human ingenuity but low belief in climate change, linked to dominion theology. Denominational differences also indicate important differences relating to perceived efficacy, with conservative Protestantism being associated with lower perceived efficacy, while Catholicism correlates with higher efficacy (Nie, 2019).

### 2.3 Eschatology

Theological factors exogenous to anthropocentrism, which may nonetheless be adjacent in character, include eschatology and perspectives on human agency/determinism. Eschatology refers to the study of “the final end of things, the ultimate resolution” of existence (Walls, 2009), with explicit perspectives being prevalent in many religions across the world. This has potential relevance to climate and environmental perspectives, with climate change in particular theorised as a secular or green eschatology (Kuehn, 2019; Northcote, 2015; Cochet, 2015). This emphasises that while this factor may not solely be a function of religion, as noted, evidence of implicit cultural influences from religious institutionalisation (Tyson, 2021) may be relevant in influencing contemporary perspectives on climate change, including its framing as an existential threat. Indeed, Christian scripture is laden with descriptions and prophecies of apocalypse, including the great flood and the burning of Sodom and Gomorrah. These descriptions directly implicate weather and climate events, with scholars emphasising their power in affecting Christian perspectives towards the environment (Curry, 2008; Guth et al., 1995).

Empirical evidence regarding eschatology and climate attitudes is mixed. Lowe et al.’s (2023) study of predominantly young Christian evangelical undergraduates emphasise the complexities of studying these perspectives, with apocalypse representing for these individuals a possibility for renewal, rather than outright destruction. Furthermore, the students generally have pro-environmental and climate attitudes. Therefore, while there is evidence that eschatological conceptualisations are important in guiding perspectives on the environment, how these perspectives influence specific climate change beliefs remains an underexplored question.

**Figure 2:**
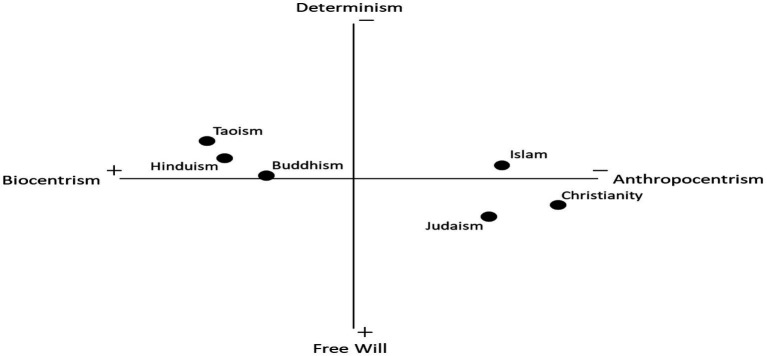
Two-dimensional plot representing the typology of religion relating to its expected effect on climate change risk perception.

Our theoretical framework is summarised in a conceptual model (Figure 2), and it predicts that Christians and Christianity are generally expected to indicate the lowest risk perception, followed by the other Abrahamic religions—Judaism and Islam. Amongst Christians, Protestants are expected to have a lower risk perception than other denominations. Buddhists and Buddhism are generally expected to have a higher risk perception. Due to the nature of cross-national research, it is difficult to determine this precisely, as individual and country-level differences vary substantially. Nonetheless, this theoretical framework provides a foundation through which individual religions can be differentiated for environmental research. The free will/determinism axis and eschatology are not included in the survey data; however, they are considered indispensable for outlining the theoretical influence of religion and religiosity on climate change perceptions. Below, individual and country-level factors are explored in more detail.

### 2.4 Attendance of religious services

Further factors include religious affiliation, attendance, practices and beliefs. These elements have been explored across studies to varying extents. Attendance of religious services is expected to act as a mechanism for more consistent beliefs amongst adherents, with strong in-group perspectives that are likely to differ from the general population. It is expected that individuals who attend regularly are likely to differ from both non-religious and similarly affiliated religions individuals who do not attend regularly. However, the perspective amongst religious attendees is also likely influenced by the characteristics and beliefs of the specific religion in question. Therefore, the effect of religious attendance is also likely to vary substantially across religions, with the expectation that Christians who attend regularly are likely to have lower risk perception than the general population, while Buddhists are expected to have higher risk perception.

Attendance and affiliation are not perfect proxies for religious practice, due to varying socio-cultural importance and purposes across countries and traditions. For example, in Japan, attendance of religious services and affiliation remain high, despite widespread “religious indifference” noted explicitly by Japanese high and supreme courts (Nelson, 2012). Furthermore, conceptualisations of religious as “perceived” before “believed” and “felt” rather than “thought” (Picken, 1994) coincide with cross-denominational identification and low levels of “personal belief in religion” at only 10.56% in 2005 (Kimpara, 2015). These limitations are discussed explicitly in the context of the data used in this research in the Methodology section.

### 2.5 Fundamentalism and literalism

Studies generally find that religious fundamentalism negatively predicts pro-environmental attitudes and climate change risk perception (Preston and Shin, 2022), with fundamentalism—alongside spirituality—proving a better predictor than general religiosity. This suggests that an international analysis of fundamentalism may yield valuable insights into climate risk perception, particularly within a comparative framework pertaining to several religions. While doctrines differ substantially, the commonality amongst fundamentalist believers in scripture as inerrant can manifest similarly across religions, often coinciding with authoritarian beliefs, structures and restrictive social orders (Altemeyer and Hunsberger, 2005). This emphasises that interpreting fundamentalism solely through the lens of scripture is likely to be overly reductive; cultural, political, and psychological factors must also be considered.

Religious fundamentalism is consistently associated with authoritarianism and social conservatism (Hunsberger, 1995), traits that mediate its relationship with environmental concern (Skalski et al., 2022). Importantly, manifestations of fundamentalism span major world religions—including Christianity, Islam, Hinduism, Buddhism, and Judaism—implying no religion is immune, regardless of its scriptural tone. However, research on non-Abrahamic fundamentalisms remains limited, and environmental perspectives may nonetheless differ in quality depending upon the specific scripture.

While the effect of fundamentalism on climate perceptions may vary by religion, its overall influence is likely negative when compared to secular or liberal expressions of the same faith. For instance, Christian fundamentalists are expected to show lower risk perception than the general population, while Buddhist fundamentalists may display higher concern—though this remains speculative. Ultimately, fundamentalism is conceptualised as a secondary factor, like religious attendance, with its effect potentially contingent on how a religion frames environmental issues. Hence, while fundamentalism is a likely contributor to climate attitudes, its precise role remains open to empirical analysis.

### 2.6 Religiosity and wealth

In high-income nations, declining religiosity has been linked to economic development, social stability, and prosperity (Barro and McCleary, 2003; Storm, 2017; Baar, 2021). The functionalist perspective of religion proposed within the secularisation thesis (Zheng et al., 2020; Sachdeva, 2016) posits that as material needs are increasingly met through state institutions, the functional necessity of religion diminishes. In contrast, in less wealthy countries, religion may retain greater importance—both as a social glue and as a framework for interpreting complex issues like climate change, especially where scientific infrastructure is limited. Religion can thus serve as both a source of morality and a relatively stable epistemological framework. In lower-income countries, this may heighten climate concern through stewardship or justice-oriented teachings. Furthermore, it may act as an adaptive coping strategy in the face of climate uncertainty, emphasising its functionalist role. In wealthier countries, however, religion may be associated with lower climate risk perception, where climate perceptions are shown to rely upon trust in scientific institutions.

Despite these dynamics, little research has explored how the same religious affiliation may function differently across varying levels of wealth. While some studies have examined religion’s effect on economic growth (Wang and Lin, 2014; Ruck and Lawson, 2018), few have addressed how wealth conditions shape religious ideology and its impact on climate perceptions. Understanding this relationship is essential for developing context-sensitive climate communication strategies and for appreciating how material and ideological conditions co-produce environmental attitudes.

## 3 Methodology

This study analyses data from 28 countries using random intercept and random slope multilevel models (Snijders and Bosker, 2012; Heisig and Schaeffer, 2019) using Stata 19 to examine individual and cross-national variation in risk perception.

The models incorporate both individual-level variables (e.g., religious affiliation, attendance of religious services, age) and country-level variables (e.g., majority religion, GDP per capita). The ability to explore the effect of both individual and country-level religion is crucial. Much of the evidence on climate change risk perception summarised above relates to the effect of individual-level religion. Yet research also suggests that, at the country level, “historical manifestations of religious nationalism and a close and supportive relation between state and dominant church increase the salience of religious boundaries,” even after accounting for levels of secularisation (Trittler, 2017). This indicates that the historically dominant religion remains integral to a country’s institutional structure, despite widespread secularisation across many regions (Tyson, 2021). This is expected to function similarly to Protestant ideology in Weber’s (1992) theory outlined in The *Protestant Ethic and the Spirit of Capitalism*. Therefore, it is expected that interpretations of climate change and environmental issues are likely shaped by a variety of social institutions, many of which have been historically—and in some cases, continue to be—shaped by religious influence (Desbordes and Munier, 2021). The implicit cultural influence of religion is likely to be captured by a country’s majority religion, which may not correspond to its standing as a majority or plurality demographic on the individual level. For example, China has rapidly secularised due to economic development and direct government intervention. Nonetheless, its historical roots in Buddhist and Daoist theology are expected to retain some influence on Chinese climate change discourses and initiatives.

Random slopes are included for individual-level religious affiliation and attendance of religious services, enabling analysis of the varying effects of these factors across countries.

To explore how the effect of religion varies across countries of differing levels of wealth, we also fit cross-level interactions between the individual-level religion variables and GDP per capita.

### 3.1 Dataset

**Figure 3:**
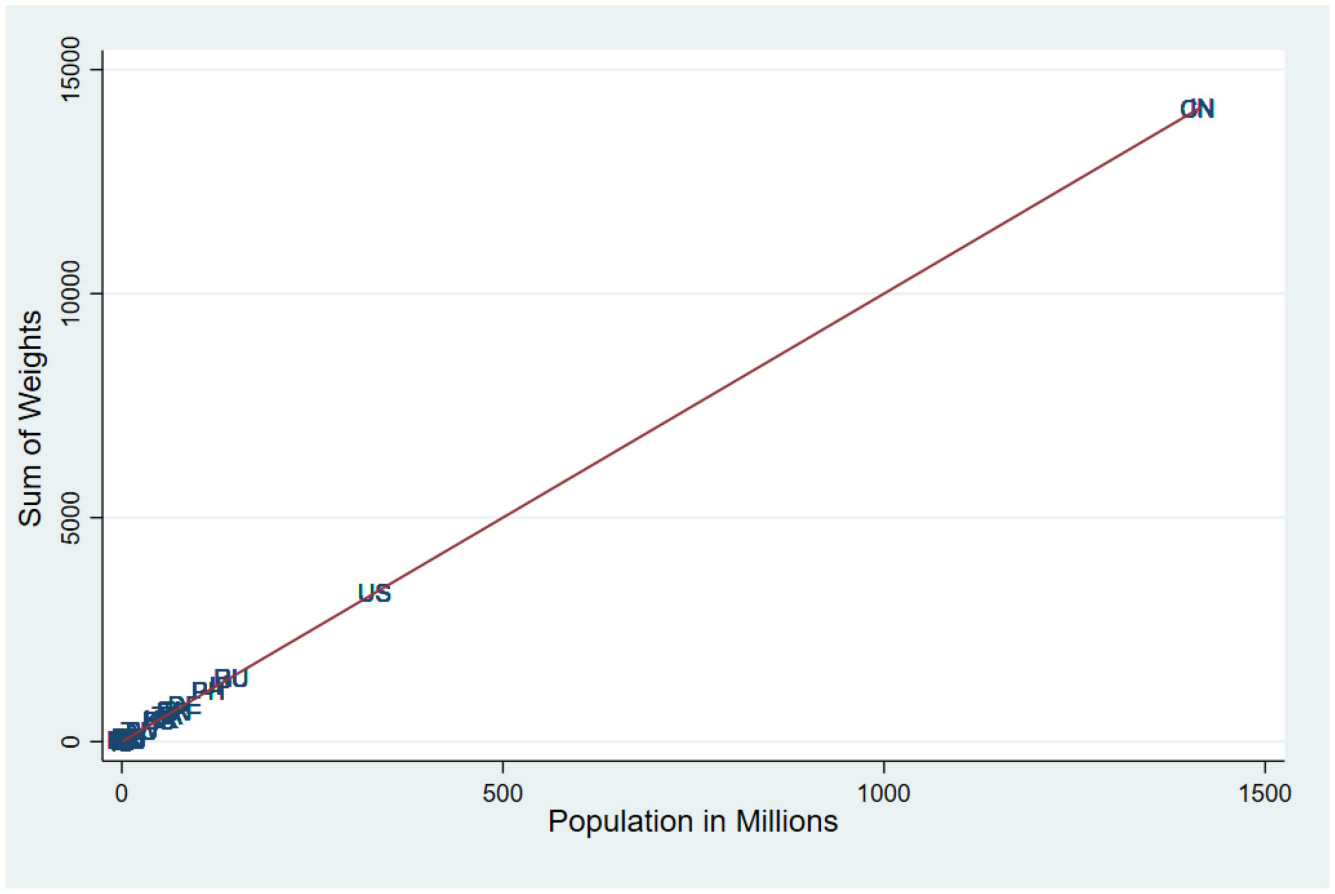
Countries by sum of weight and population size.

The analysis draws on the ISSP “environment” IV survey (GESIS, 2023; Hadler, 2019), which includes nationally representative samples from 28 countries. Each country’s data was collected by local teams, with minor methodological differences, and later standardised by the International Social Survey Programme (ISSP). Data collection occurred between October 15, 2019, and May 31, 2023—a timeline extended due to delays from the COVID-19 pandemic. Sample sizes ranged from 1,000 to 4,280 respondents per country (mean = 1,575). The weighting procedures across countries varied somewhat, with some countries not including weights (GESIS, 2023). This was addressed by dividing 1 by the number of respondents and applying the figure to each respondent. These weights were applied to all analyses to ensure representativeness and scaled to adjust for differences in population size across countries (Carle, 2009), as shown in Figure 3.

The multilevel random-effects structure is in line with methodological literature that has reviewed the robustness of multilevel models for national comparison. For example, Bryan and Jenkins (2016) recognised the advantages of multilevel for multivariate cross-country comparison and recommended that a minimum of 25 countries be included to provide robust country-level effects. Multilevel modelling has become an established tool for testing cross-national comparison hypotheses, while controlling for underlying demographic country differences (Fairbrother, 2014; Franzen and Vogl, 2013; Hick et al., 2025).

### 3.2 Measures

Climate change risk perception was assessed using two similarly worded survey items from the ISSP environment IV module (Hadler, 2019).

- World risk perception was measured with the question: “On a scale from 0 to 10, how bad or good do you think the impacts of climate change will be for the world as a whole?” (0 = extremely bad, 10 = extremely good).
- Country risk perception used the same format but replaced “the world as a whole” with the respondent’s own country.

These two measures were included to capture different forms of risk perception—global and national—and to assess whether the influence of explanatory variables differs depending on the perspective taken. These scales were reversed prior to analysis so that 10 indicates high risk perception (“extremely bad”).

Individual-level religious affiliation was measured firstly through country-specific questionnaires and harmonised by the ISSP into a comparative category to allow for cross-country analysis. Attendance of religious services is based on answers to the question: *“Apart from such special occasions as weddings, funerals and baptisms, how often nowadays do you attend services or meetings connected with your religion?.”* The answers were recoded from eight to three categories, including ‘Never’, ‘Less than once a month’, and ‘More than once a month’. Lemos et al. (2019) noted the limitations of the ISSP religion survey, of which only a small proportion of the total questionnaire is included in the environmental survey, in terms of establishing metric invariance. This is a particularly important consideration for cross-national comparative research, as noted above. This research recognises the limitations of the metrics used, particularly attendance acting as the singular variable for religious practice, but emphasises its importance in expanding discussion and empirical evidence in an underexplored field.

Control variables include political affiliation, perceived efficacy (personal and collective), national priorities, sex, age, income, and education. The sociodemographic factors and politics are included in the CCRPM (Van Der Linden, 2015). Perceived efficacy measures are also considered impactful contributors to risk perception (Brody et al., 2008; Kellstedt et al., 2008; Capstick and Pidgeon, 2014).

The postmaterialism variables are based on two questions asking about their perceived priorities for the country, which is devised as a 4-point index to measure postmaterialism (Inglehart, 1977). Individuals who chose both “protect freedom of speech” and “give people more say in government decisions” are categorised as postmaterialists. Individuals who chose both “maintain order in the nation” and “fight rising prices” are categorised as traditionalists. Individuals who chose options from both categories, i.e., maintain order in the nation and protect freedom of speech, are categorised as neither. These measures are included to characterise varying value orientations within political groups, sometimes labelled as “old” (materialist) and “new” (postmaterialist) left/right in sociological literature (Kaltenthaler et al., 2008).

Personal efficacy is based on respondent’s level of agreement with the statement “It is just too difficult for someone like me to do much about the environment.” It is measured on a 1–5 Likert scale ranging from “strongly disagree” to “strongly agree”. Collective efficacy is based on the statement ‘There is no point in doing what I can for the environment unless others do the same’, with the same responses on a Likert scale. Efficacy measures are included to understand the impact of an individual’s perceived control on their climate risk perception within society, which is a different conceptualisation from control relating to free will and determinism. Sensitivity analysis indicates that the results remain unchanged regardless of the inclusion of these measures.

Aggregate or country-level variables were also included. The majority religion and the proportion of Christians are two aggregate-level measures of religion. The majority religion was measured with the following two categories: Christian and Eastern Religions. Eastern Religions were combined into one category due to the small number of countries and similarly lower emphasis on anthropocentrism in comparison to Christianity. The category contains Buddhism, Daoism, Shintoism, and Hinduism. An alternate majority religion variable, coded as Protestant, Other Christian, and Eastern Religions, was also tested alongside added control variables (emissions, Climate Risk Index, Climate Change Performance Index) (see Appendix M5-M7) to further test the robustness of the main findings. GDP per capita was included as a log of GDP.

Descriptive statistics, including missingness rates for all measures, are included in the Appendix (Supplementary Tables A.1, A.2). Given low levels of missingness, listwise deletion was used. A variance component model is included to allow for ICC comparison with the models (Supplementary Table A.3). Sensitivity analysis was also performed including tests for multicollinearity (i.e., variance inflation factor (VIF) Supplementary Table A.4). These tests showed low-to-moderate collinearity, with a mean VIF of 1.61 for country risk and 1.62 for world risk, with no values exceeding 5.

## 4 Results

This section presents the results from the multilevel random-intercept (Models 1 and 2) and random-slopes models (Models 3 and 4), with models differentiated by the inclusion of majority religion and proportion of Christians, followed by random slopes for attendance of religious services. Model diagnostics are reported at the bottom of each table, including Level-1 and Level-2 *R*^2^ values, Intraclass Correlation Coefficients (ICCs), and Akaike information criterion/Bayesian Information Criterion (AIC/BIC) model fit indices (Snijders and Bosker, 2012). The appendix contains sensitivity analyses using additional aggregate variables, and survey year (Supplementary Tables A.5, A.6), as well as alternate variables to GDP (log)—HDI and GDP (PPP) (Supplementary Table A.7)—along with descriptive statistics. All models reported VIFs below 5, with mean VIFs of 1.61 for country risk and 1.62 for world risk (Supplementary Table A.4). Ordinal logistic models yielded results consistent with the linear models presented below (see Supplementary Table A9 and note). We also found consistent results when we re-ran the models with collapsed attendance and affiliation categories (see Supplementary Table A.8 and Supplementary Figure A.1 in the Appendix). Additional analyses (not shown) addressed heteroskedasticity concerns (see Table 1).

**Table 1:** Random intercept and slope models for major religion and attendance of religious services.

Predictors	Country risk (M1)	World risk (M1)	Country risk (M2)	World risk (M2)	Country risk (M3)	World risk (M3)
Political affiliation (center/center-left)						
Center left	0.22 (0.13)	0.16 (0.13)	0.22 (0.13)	0.16 (0.13)	0.22 (0.13)	0.16 (0.13)
Left/far left	0.42* (0.11)	0.42* (0.12)	0.43* (0.11)	0.42* (0.12)	0.41* (0.11)	0.42* (0.12)
Center right	−0.26 (0.15)	−0.34* (0.15)	−0.26 (0.15)	−0.34* (0.15)	−0.26 (0.15)	−0.34* (0.15)
Right/far right	−0.39* (0.19)	−0.43* (0.21)	−0.39* (0.19)	−0.44* (0.21)	−0.40* (0.19)	−0.43* (0.21)
Refused/didn’t vote	−0.02 (0.10)	−0.04 (0.10)	−0.02 (0.10)	−0.04 (0.10)	−0.02 (0.10)	−0.04 (0.10)
Religion (no religion)						
Catholic	−0.05 (0.07)	−0.02 (0.06)	−0.04 (0.07)	−0.01 (0.06)	−0.05 (0.07)	−0.02 (0.06)
Protestant	−0.12 (0.10)	−0.13 (0.11)	−0.12 (0.10)	−0.13 (0.11)	−0.10 (0.10)	−0.13 (0.11)
Orthodox	−0.17 (0.15)	−0.25 (0.18)	−0.16 (0.15)	−0.24 (0.18)	−0.16 (0.15)	−0.25 (0.18)
Other Christian (Varies by country)	−0.15 (0.11)	−0.20 (0.12)	−0.15 (0.11)	−0.20 (0.12)	−0.10 (0.11)	−0.20 (0.12)
Jewish	0.14 (0.25)	0.10 (0.34)	0.15 (0.25)	0.10 (0.34)	0.15 (0.25)	0.10 (0.34)
Islamic	−0.16 (0.19)	−0.22 (0.15)	−0.16 (0.19)	−0.20 (0.15)	−0.16 (0.19)	−0.22 (0.15)
Buddhism	0.17 (0.15)	0.18 (0.13)	0.15 (0.15)	0.18 (0.13)	0.11 (0.15)	0.18 (0.13)
Hindu	0.05 (0.23)	−0.15 (0.16)	0.04 (0.23)	−0.07 (0.16)	0.07 (0.23)	−0.15 (0.16)
Other Asian Religions (Varies by country)	0.00 (0.10)	−0.04 (0.06)	0.01 (0.10)	−0.04 (0.06)	0.05 (0.10)	−0.04 (0.06)
Other (Varies by country)	0.18 (0.16)	0.31 (0.17)	0.18 (0.16)	0.31 (0.17)	0.17 (0.16)	0.31 (0.17)
Attendance of Religious Service (Never)						
Once a month or less	−0.13* (0.04)	−0.13* (0.04)	−0.13* (0.04)	−0.13* (0.04)	−0.16* (0.04)	−0.15* (0.04)
More than once a month	−0.27* (0.09)	−0.31* (0.09)	−0.27* (0.09)	−0.30* (0.09)	−0.31* (0.09)	−0.30* (0.08)
National Priorities (Materialist)						
Postmaterialist	0.08 (0.07)	0.15* (0.07)	0.08 (0.07)	0.15* (0.07)	0.08 (0.07)	0.15* (0.07)
Neither	−0.06 (0.04)	−0.01 (0.05)	−0.06 (0.04)	−0.01 (0.05)	−0.06 (0.04)	−0.01 (0.05)
Personal Efficacy	0.12* (0.03)	0.13* (0.03)	0.12* (0.03)	0.13* (0.03)	0.12* (0.03)	0.13* (0.03)
Collective Efficacy	0.12* (0.02)	0.14* (0.03)	0.12* (0.02)	0.14* (0.03)	0.12* (0.02)	0.14* (0.03)
Age	−0.01* (0.00)	−0.01* (0.00)	−0.01* (0.00)	−0.01* (0.00)	−0.00* (0.00)	−0.01* (0.00)
Sex (Male)	0.14* (0.04)	0.11* (0.03)	0.14* (0.04)	0.11* (0.03)	0.14* (0.04)	0.11* (0.03)
Education (Upper Secondary)						
No Education	−0.03 (0.17)	0.06 (0.13)	−0.03 (0.17)	0.07 (0.13)	−0.03 (0.17)	0.06 (0.13)
Primary	0.04 (0.07)	0.05 (0.09)	0.04 (0.07)	0.06 (0.09)	0.04 (0.07)	0.05 (0.09)
Lower secondary	−0.01 (0.05)	0.00 (0.05)	−0.01 (0.05)	0.01 (0.05)	−0.02 (0.05)	0.00 (0.05)
Post-secondary/short-cycle tertiary	0.03 (0.05)	0.05 (0.06)	0.03 (0.05)	0.05 (0.06)	0.03 (0.05)	0.05 (0.06)
Lower tertiary (BA)	0.17* (0.04)	0.18* (0.05)	0.16* (0.04)	0.17* (0.05)	0.17* (0.04)	0.18* (0.05)
Upper tertiary (MA)	0.28* (0.07)	0.37* (0.09)	0.28* (0.07)	0.37* (0.09)	0.28* (0.07)	0.37* (0.09)
Post-tertiary (PhD)	0.46* (0.12)	0.67* (0.09)	0.46* (0.12)	0.67* (0.09)	0.47* (0.12)	0.67* (0.09)
Income (25% quartile)						
Lower middle	−0.02 (0.03)	0.07 (0.04)	−0.02 (0.03)	0.07 (0.04)	−0.02 (0.03)	0.07 (0.04)
Upper middle	0.03 (0.05)	0.12* (0.05)	0.03 (0.05)	0.12* (0.05)	0.03 (0.05)	0.12* (0.05)
Upper quartile	−0.00 (0.07)	0.11 (0.07)	−0.00 (0.07)	0.11 (0.07)	−0.00 (0.07)	0.11 (0.07)
No answer/refused	0.01 (0.06)	0.03 (0.06)	0.01 (0.06)	0.03 (0.06)	0.01 (0.06)	0.03 (0.06)
Major religion (simplified)						
Eastern religion	0.55* (0.22)	0.38 (0.25)			0.41* (0.15)	0.38 (0.25)
Percentage of Christians			−0.01* (0.00)	−0.01* (0.00)		
GDP (Log)	0.13 (0.11)	0.34* (0.10)	0.08 (0.09)	0.31* (0.08)	0.16 (0.13)	0.34* (0.10)
Government restriction	−0.16 (0.10)	−0.09 (0.09)	−0.15 (0.09)	−0.09 (0.09)	−0.24 (0.15)	−0.09 (0.09)
Social hostility	0.10 (0.11)	0.09 (0.10)	0.05 (0.11)	0.06 (0.09)	0.10 (0.11)	0.09 (0.10)
Constant	4.61* (1.18)	2.85* (1.08)	5.78* (0.92)	3.66* (0.85)	4.92 (1.54)	2.67 (1.91)
Var (Constant)	0.21 (0.07)	0.16 (0.04)	0.17 (0.06)	0.14 (0.04)	0.32 (0.10)	0.31 (0.01)
Var (Residual)	4.80 (0.24)	4.95 (0.27)	4.80 (0.24)	4.95 (0.27)	4.78 (0.24)	4.94 (0.27)
Var (Attendance—Once a month or less)					0.16 (0.04)	0.16 (0.04)
Var (Attendance—More than once a month)					0.38 (0.07)	0.35 (0.07)
Observations (Countries)	34,305 (28)	34,353 (28)	34,305 (28)	34,353 (28)	34,305 (28)	34,353 (28)
ICC	0.04	0.03	0.03	0.03	0.04	0.04
Snijders/Bosker R-squared Level 1	0.04	0.06	0.05	0.07	0.04	0.06
Snijders/Bosker R-squared Level 2	0.27	0.49	0.36	0.55	0.27	0.49
AIC	128018.9	129182.2	128015.1	128178.9	128179.6	129299.7
BIC	128365.1	129528.4	128361.2	129525.1	128534.2	129662.5

*p < 0.05.

Standard errors are reported in brackets unless labelled otherwise.

No nonconcavity, boundary issues or integration accuracy problems were encountered. All models converged within eight iterations. All variance components were above zero.

Model 1 indicates that climate change risk perception decreases with more frequent attendance of religious services. Those who attend somewhat frequently (once a month or less) have 0.13 points lower country and world risk perception than those who never attend. Those who attend very frequently (more than once a month) have a lower risk perception still (0.27, 0.31). Individual religious affiliation has no statistically significant effect, and this is true when the efficacy measures are removed too. Individuals living in Eastern religious majorities (Buddhist and Hindu), on average, have a 0.55 higher country risk perception than those living in Christian-majority countries. As expected, the Level-1 *R*^2^ is low, explaining 4 and 6% of the variation at the individual level. However, the Level-2 *R*^2^ shows substantial explanatory power at the country level, explaining 27 and 49% of the variation.

Model 2 replaces the majority religion effect in M1 with a percentage Christian one. It shows that the proportion of Christians significantly predicts both country and world risk perception, with 0.01 lower risk perception for each percentage point more Christians in a country. For example, if a country has 25% Christians, individuals living in that country will, on average, have a 0.50 higher risk perception than individuals living in a country of 75% Christians. Individual religious affiliation and aggregate measures for fundamentalism indicate no significant effects on risk perception across all models. The Level-2 *R*^2^ is higher in this model for both Country Risk Perception (CR) (36%) and World Risk Perception (WR) (55%).

**Figure 4:**
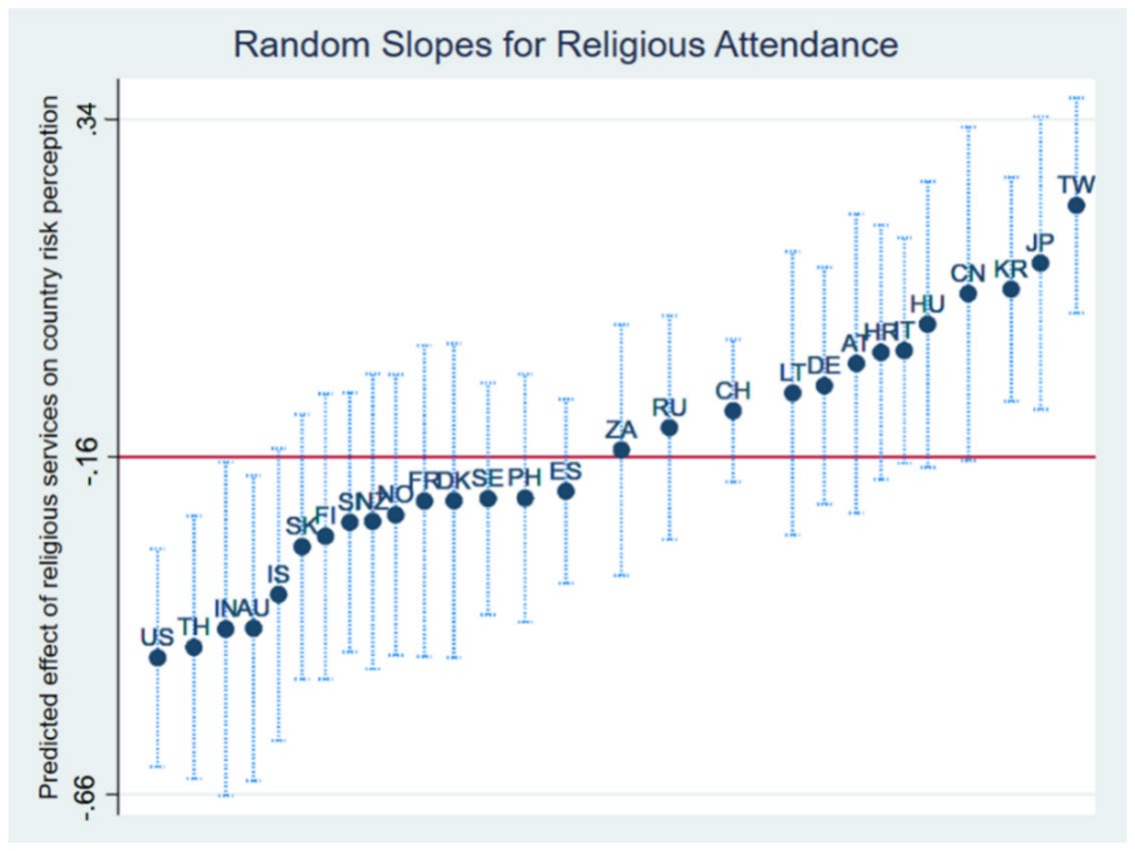
Predicted slopes for attendance of religious services on country risk perception across countries (M3).

Model 3 includes random slopes for attendance of religious services. The models indicate variation across countries in terms of the effect of attendance of religious services on risk perception. The majority indicate a negative effect for attendance of religious services, with Buddhist-majority countries, in addition to Thailand, all indicating either the smallest negative effects or slightly positive effects, observable in Figure 4. Furthermore, the USA and Australia both indicate some of the largest negative effects of attendance of religious services on risk perception (see Table 2). Christian-majority countries generally ind icate negative effects. The effect for the majority religion persists but is slightly smaller than Model 1 (0.41)^1^.

**Table 2:** Random slopes with cross-level interactions for religious affiliation and country wealth.

Predictors	Country risk (M4)	World risk (M4)
Religious affiliation (no religion)		
Catholic	3.79* (1.37)	4.39* (1.28)
Protestant	4.20* (1.52)	5.18* (1.53)
Orthodox	4.48* (1.15)	4.19* (1.09)
Other Christian	4.31* (1.56)	6.02* (1.72)
Jewish	−2.89 (6.63)	−0.63 (6.77)
Islamic	4.16* (1.15)	4.52* (1.36)
Buddhist	4.62* (2.29)	4.37 (2.41)
Hindu	3.22 (2.45)	4.08* (1.01)
Other Asian religions	−0.11 (2.44)	3.86* (1.61)
Other religions	2.31 (1.23)	5.14* (1.23)
GDP (Log)	0.42* (.12)	0.68* (0.11)
Religious affiliation#GDP		
Catholic	−0.36* (0.13)	−0.41* (0.12)
Protestant	−0.40* (0.14)	−0.49* (0.16)
Orthodox	−0.46* (0.12)	−0.43* (0.11)
Other Christian	−0.43* (0.15)	−0.60* (0.17)
Jewish	0.27 (0.60)	−0.06 (0.60)
Islamic	−0.41* (0.11)	−0.45* (0.14)
Buddhist	−0.44 (0.23)	−0.40 (0.23)
Hindu	−0.37 (0.24)	−0.38* (0.11)
Other Asian religions	−0.03 (0.23)	−0.36* (0.15)
Other religions	−0.20 (0.12)	−0.46* (0.11)
Constant	0.68	−2.23
Var (Catholic)	0.13	0.12
Var (Protestant)	0.26	0.23
Var (Orthodox)	0.43	0.25
Var (Other Christian)	0.28	0.36
Var (Jewish)	1.87	6.47
Var (Islamic)	0.63	0.76
Var (Buddhist)	0.65	0.96
Var (Hindu)	3.99	3.07
Var (Other Asian religions)	3.09	3.79
Var (other religions)	0.40	0.25
Var (constant)	0.37	0.25
Var (residual)	4.02	4.15
Observations (countries)	34,305 (28)	34,353 (28)
ICC	0.08	0.07
Snijders/Bosker *R*-squared Level 1	0.04	0.07
Snijders/Bosker *R*-squared Level 2	0.25	0.51
AIC	127968.6	129133.9
BIC	128407.7	129573

*p < 0.05.

Controls include all individual-level variables from M1 as well as all country-level variables except the majority religion (simplified).

**Figure 5:**
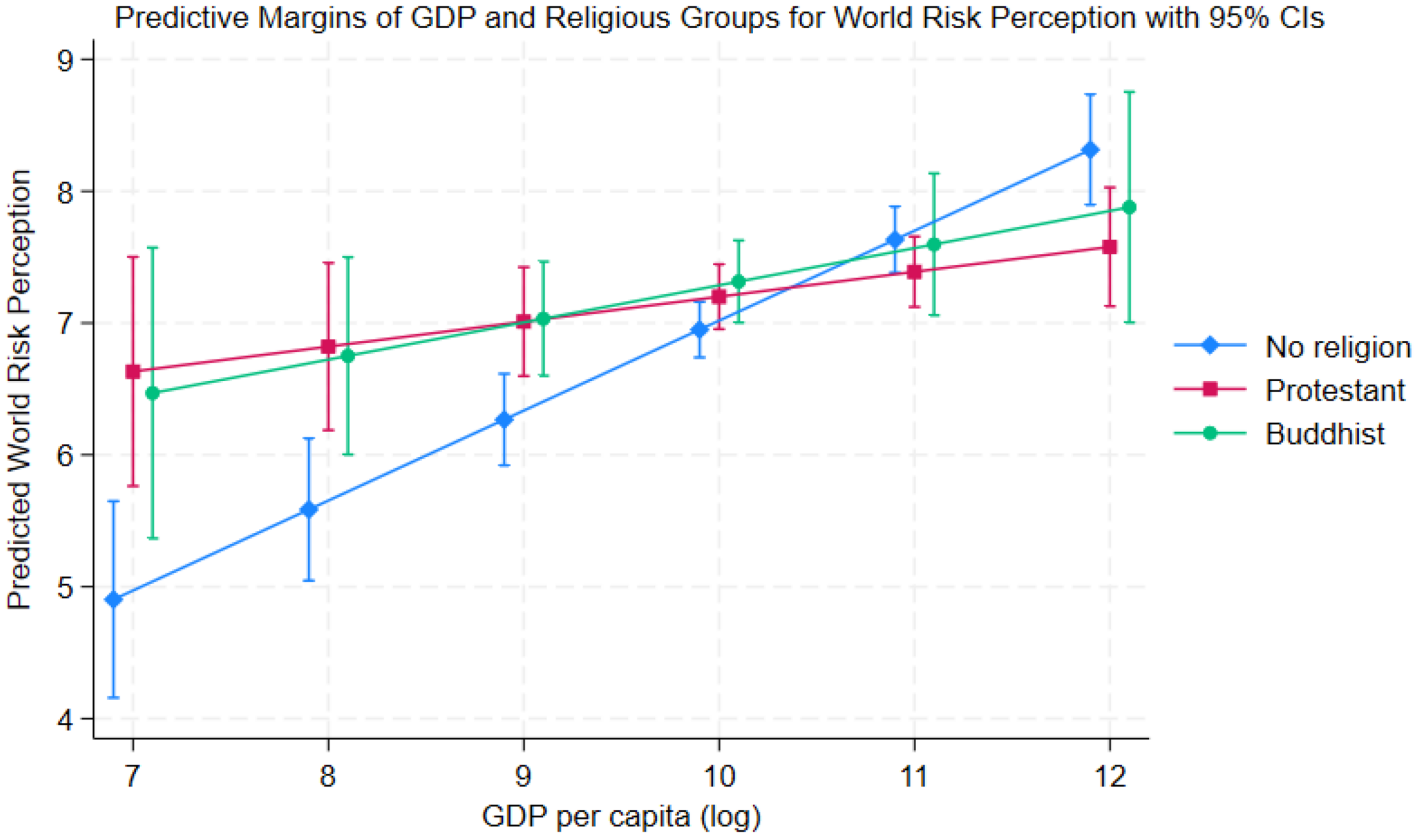
Cross-level interaction between GDP per capita and individual-level religious affiliation on world risk perception (M4).

Model 4 includes the cross-level interaction for GDP and religious affiliation, indicating that GDP per capita moderates the effect of religious affiliation on climate change risk perception. Religiously affiliated individuals generally have higher country and world risk perception than non-religious individuals in low-income countries^2^, as observed in Figure 5. However, as in wealthier countries, this gap closes with similar levels of risk perception in middle- and high-income countries. In high-income countries, individuals affiliated with Protestant, Catholic, Orthodox, Other Christianity, and Islam indicate lower country risk perception in comparison to non-religious individuals. The same goes for these religions and Hinduism for world risk perception.

This shows a reversal of the effect observed in low-income countries. For example, Orthodox Christians in countries with a GDP per capita lower than $16,305 would indicate higher risk perception than non-religious individuals in the same country. However, in countries where GDP exceeds this figure, non-religious individuals would be expected to have the same or higher country risk perception across the religions noted above. The effect is similar for world risk perception, too, with a more pronounced slope for non-religious individuals (Figure 5).

## 5 Discussion

Although it is challenging to directly connect the theoretical constructs of anthropocentrism and free will to climate change risk perception, the findings align with the proposed typology, suggesting that Christianity, at the national level, is associated with lower perceived climate risks than religions, such as Buddhism, Daoism, and Hinduism. Future research could usefully explore this dynamic in the context of other Abrahamic faiths, particularly Islam and Judaism. However, the current study was limited in this regard due to the absence of countries with majority Muslim or Jewish populations in the sample.

Moreover, the theoretical proposition that climate change may be interpreted through apocalyptic or other religious narratives found limited empirical support. This is especially evident in the relatively weak influence of religious variables on perceptions of world risk, which was an outcome that would be expected to align most closely with apocalyptic perspectives. These findings underscore the need for a more nuanced understanding of the mechanisms underlying religious influences on climate risk perception. Given the probable complexity of any causal relationships (Curry, 2008; Eckberg and Blocker, 1996), future research would benefit from incorporating survey measures addressing beliefs in free will, anthropocentrism, and other qualitative dimensions of religious philosophy.

### 5.1 Attendance of religious services

The negative effects of religious attendance across the 28 countries offer an indication that the consistency of religious practice is an important consideration when analysing the effects of religiosity. While individuals may be associated with a religion, it is the engagement with practices which, depending upon the religion, may expose individuals to theological perspectives wherein environmental and climate perceptions may be impacted. The effect of religious services is generally negative across the countries, though the random slopes indicated some variation, which corresponds with the typologies of major religions to a degree. The risk perception of individuals in Taiwan, Japan, Korea, and China is least negatively impacted by attendance of religious services, with all of these countries being Buddhist-majority countries. Meanwhile, the USA and Australia indicate large negative effects for attendance of religious services. The literature has explored churchgoers in both the USA (Arbuckle, 2017) and Australian populations (Pepper and Leonard, 2016; Tranter and Booth, 2015), indicating that these demographics tend to have higher climate scepticism.

This could be indicative of the importance of ideological variation across theological traditions and supports the notion that exposure to this variation is captured more by frequency of practice over affiliation. As noted, White (1967) placed emphasis on the anthropocentric nature of Christianity and the earlier association of Christian countries with capitalist resource extraction. On the other hand, Buddhism and Hinduism place more explicit emphasis on harmonisation between humanity and the environment within scripture, which has remained relevant to the institutions of these countries, despite the prevalence of globalised capitalism in these countries too (Rishi and Schleyer-Lindenmann, 2021). Nonetheless, the breadth of ideological diversity within religions is of note too, emphasised by the lack of a substantial relationship between religious affiliation and climate change risk perception. Pepper and Leonard (2016) indicated that the specific denominations identified as most sceptical in Australia, specifically Australian Pentecostals, remain more closely identified with dominion ideology, which, as noted, contrasts with conceptions of stewardship, instead emphasising the productive capacities of humanity, offering a far more anthropocentric view than other Christian denominations. The literature from the USA has similarly emphasised the role of dominionism in explaining the generally negative attitudes towards climate initiatives in the country. This provides evidence that the variation in perspectives on anthropocentrism and biocentrism may be partly indicative of the varied effects of religious practice across countries, though this requires further research with explicit operationalisation and measurement of these concepts.

### 5.2 Religious affiliation

As noted, the effects of individual religious affiliation are minimal across the religions included. This may be due to the fact that affiliation alone does not necessitate theological consistency amongst individuals in the same religion. The low levels of attendance observable across most countries, particularly wealthier countries, in addition to the USA, emphasises a corresponding factor to that relating to the negative effect of attendance. While attendees are shown to diverge from those who do not attend, the range of perspectives within a religious group itself is likely vast, with both liberal and conservative adherents to a faith being commonplace, particularly in secularised societies where other ideological and cultural factors may be more impactful in determining individual perspectives. Therefore, the differences between non-religious and religiously affiliated individuals in these societies may be generally minor. Meanwhile, those who attend offer a unique religious perspective, which nonetheless also appears to be influenced by the specific doctrine prescribed by each denomination.

With the inclusion of the cross-level interaction for GDP, religious affiliation does exert notable effects on risk perception, which may be evidence of a ‘dual-effect’ of religion. The higher risk perception among religious individuals in low-income countries than among non-religious individuals may be indicative of the philosophical and epistemological framework that a religion is able to provide. The environmental perspectives found across most religions likely influence a level of awareness and consideration amongst adherents, and this is supported by the relatively stable risk perception identified between individuals in the same religions across countries of varying wealth. This also aligns with functionalist perspectives of religion, where the necessity and utility of religious perspectives are deemed greater in lower-income countries (Zheng et al., 2020; Sachdeva, 2016).

Meanwhile, the effect of living in a wealthier country for non-religious individuals is quite substantial, with a higher risk perception in higher-income countries. Individuals in these countries are likely to have greater exposure to scientific advances and access to higher education institutions, both of which are indicators of higher risk perception. Greater trust regarding the scientific consensus of climate change is also associated with exposure to science, which is more common in higher-income countries (Krugly-Smolska, 2007, 2007). On the other hand, lower variation amongst religious individuals likely emerges due to a greater proclivity towards scientific scepticism and lower scientific literacy in comparison to non-religious individuals in the same country (McPhetres and Zuckerman, 2018; Upenieks et al., 2021). This points towards the dual effect of religion, where the epistemological framework deemed beneficial in low-income countries may inversely impact risk perception in high-income countries.

### 5.3 Majority religion and proportion of Christians

The majority religion remains underexplored in multilevel analysis, and this research indicates that it may be a pertinent perspective to consider, particularly with the goal of capturing institutional effects of religion. The higher risk perception amongst individuals in Eastern religious-majority countries compared to Christian-majority countries, as well as Protestant-majority countries (Supplementary Table A.6 M5), is interesting. Firstly, as this is a country-level effect, it indicates that this effect occurs amongst individuals regardless of individual religious affiliation. Therefore, the institutional effect captured here indicates that the influence of the majority religion may have a potent effect on cultural discourses relating to environmental and climate issues.

The proportion of Christians living in a country also has a meaningful impact on the risk perception across countries. This also points towards the importance of the prevalence of broad ideological traditions in determining the climate perceptions of individuals in the country. However, unlike in the majority religion, the proportion of Christians is not necessarily indicative of institutional effects. This is due to the fact that large-scale immigration and the adoption of religious beliefs in a country can make demographics quite fluid, with Korea representing an example of a country where Christianity has boomed in recent decades (Baar, 2021), while having very little historical institutional influence. China is another example, where the number of religious individuals has greatly declined, yet institutional influence from Buddhism and Daoism is still observable across many elements of Chinese society.

### 5.4 Limitations and perspectives

This research aimed to contribute to a burgeoning body of literature exploring the relationship between climate change perceptions and future policy directions alongside individual and country-level religiosity (Skirbekk et al., 2020a; Chuvieco et al., 2016). The number of countries used throughout this analysis enabled an exploration of Christianity and a broader categorisation of Eastern Religions, including countries with historical ties to major religions, including Buddhism and Hinduism, as well as more geographically localised religions such as Daoism and Shintoism, amongst others. This represents a first step towards a greater understanding of the ongoing cultural legacies of these religions upon contemporary institutions in corresponding countries. However, due to the important differences between religions within this category, further research with a greater diversity of major religions across a larger set of countries would be particularly useful for expanding the discussions begun in this analysis. Additionally, case studies of countries where the major religion may not be prevalent in other countries, in terms of its majority status, such as Israel with Judaism and India with Hinduism. This evidence regarding country-level differences emphasises that this is a potentially fruitful avenue for future research.

## 6 Conclusion

This analysis of religion expands understandings of how religiosity can impact climate change risk perception from several perspectives. Firstly, the finding that attending religious services is associated with lower risk perception indicates that consistency of practice is an important vehicle for explaining varying perceptions amongst populations, even when controlling for political affiliation and beliefs of personal efficacy, and signals differences between devout religious individuals and less devout, the latter of which more closely align with non-religious individuals on the issue of climate change. Secondly, while religious affiliation is generally not a primary contributor to climate change risk perception, cross-level analysis shows that it nonetheless provides a relatively stable ideological foundation for environmental and climate risk perception, particularly in lower-income countries where non-religious individuals exhibit the lowest risk perception. In high-income countries, by contrast, the differences between religious and non-religious individuals are minor, with some denominations indicating slightly lower risk perception. Finally, Christian-majority countries have lower country risk perception than Buddhist- and Hindu-majority countries, with Protestant-majority countries consistently showing the lowest risk perception. These findings both affirm and refine the proposed theoretical framework: while the framework anticipated broad differences across religious traditions, our results indicate a particularly pronounced influence of Protestantism and highlight the dual effect of religiosity depending on national wealth. Taken together, these findings align with elements of the theoretical framework and lend further credence to White’s thesis (1967), while also extending it to contemporary cross-national contexts.

## Data Availability

All data supporting this study is available in the International Social Survey Programme data repository at: https://www.gesis.org/en/issp/data-and-documentation/environment/2020.

## Footnotes

1 We checked the variation of this coefficient using leave-one-country-out analysis, which involves re-running the models iteratively without one country and found that the coefficient had a mean close to the reported coefficient and a standard deviation of 0.05 for both country risk and world risk models (M3), which suggests that the estimate is relatively stable.

2 As these models include interaction effects, the coefficients should be interpreted differently from Models 1 to 3.
