# Supplementary material for: Towards an understanding of the impact of micro- and macro-manifestations of religiosity on climate change risk perception: a cross-national study
Source: Front Psychol. 2026 Mar 4;17:1740305. doi: 10.3389/fpsyg.2026.1740305 (PMC12998447; doi:10.3389/fpsyg.2026.1740305)
Supplement: Supplementary file 1 [file Table_1.DOCX]

**Statements and Declarations**

*“This work was supported by ESRC PhD stipend. Author R.S. has received research support from Company A.”*

*“The authors declare that no funds, grants, or other support were received during the preparation of this manuscript.”*

*“Financial interests: Author A B and C declare they have no financial interests.*

*“The authors have no relevant financial or non-financial interests to disclose.”*

*“All authors contributed to the study conception and design. Material preparation, data collection and analysis were performed by [Richard Saunders]. The first draft of the manuscript was written by [Richard Saunders] and all authors commented on previous versions of the manuscript. All authors read and approved the final manuscript.”*

“All data are available from the GESIS repository (https://search.gesis.org/research_data/ZA7650).”

“This study uses publicly available secondary data and did not require ethical approval.”
